# Ecotoxicity and Mutagenicity Assessment of Novel Antifungal Agents VT-1161 and T-2307

**DOI:** 10.3390/molecules29194739

**Published:** 2024-10-07

**Authors:** Edith Guadalupe Padilla Suarez, Antonietta Siciliano, Marisa Spampinato, Angela Maione, Marco Guida, Giovanni Libralato, Emilia Galdiero

**Affiliations:** 1 Department of Biology, University of Naples Federico II, Complesso Universitario Monte Sant’Angelo, Via Cintia 4, 80126 Naples, Italy; edith.padilla@unina.it (E.G.P.S.); marisa.spampinato@unina.it (M.S.); angela.maione@unina.it (A.M.); marguida@unina.it (M.G.); giovanni.libralato@unina.it (G.L.); egaldier@unina.it (E.G.); 2 NBFC—National Biodiversity Future Center, 90133 Palermo, Italy

**Keywords:** antifungal agents, environmental impact, ecotoxicity, VT-1161, T-2307

## Abstract

Antifungal substances are essential for managing fungal infections in humans, animals, and plants, and their usage has significantly increased due to the global rise in fungal infections. However, the extensive application of antifungal agents in pharmaceuticals, personal care products, and agriculture has led to their widespread environmental dissemination through various pathways, such as excretion, improper disposal, and agricultural runoff. Despite advances in wastewater treatment, many antifungal compounds persist in the environment, affecting non-target organisms and contributing to resistance development. This study investigates the environmental impact of two novel antifungal agents, VT-1161 and T-2307, recently introduced as alternatives for treating resistant *Candida* spp. We assessed their ecotoxicity and mutagenicity using multiple bioassays: immobilization of *Daphnia magna*, growth inhibition of *Raphidocelis subcapitata*, luminescence inhibition of *Aliivibrio fischeri*, and mutagenicity on *Salmonella typhimurium* strain TA100. Results indicate that both VT-1161 and T-2307 exhibit lower toxicity compared to existing antifungal compounds, with effective concentrations (EC_50_) causing 50% response ranging from 14.34 to 27.92 mg L^−1^. Furthermore, both agents were classified as less hazardous based on the Globally Harmonized System of Classification and Labeling of Chemicals. Despite these favorable results, further research is needed to understand their environmental behavior, interactions, and potential resistance development among non-target species. Our findings highlight the importance of comprehensive environmental risk assessments to ensure the sustainable use of new antifungal agents.

## 1. Introduction

Antifungal substances play a crucial role in treating fungal infections in humans, animals, and plants, particularly in ensuring crop growth [1,2]. The pharmaceutical industry has seen a notable increase in the use of antifungal medications, due to the increasing prevalence of fungal infections globally [3,4,5]. Moreover, these compounds are also commonly present in multiple personal care products [6,7]. Additionally, the use of fungicides in agriculture as a preventive measure has substantially increased over the last decades [8].

The widespread use of antifungal substances has resulted in various pathways for these substances into the environment, including through human and animal excretion, improper disposal of medications, and agricultural runoff [9,10,11]. Despite advancements in wastewater treatment systems, they often struggle to remove fungicides fully [7,9]. This inefficiency is attributed to variations in hydrophilicity and biodegradation rates among different compounds [9]. When released into the environment, antifungal substances can contaminate multiple matrices, including surface and groundwater, soil, and sediment [12,13].

Although antifungal drugs are designed to target fungal cells specifically, they can also impact non-target organisms in the environment, leading to adverse effects on multiple terrestrial and aquatic organisms across various trophic levels [9,14,15,16]. Due to their potential environmental impact and increasing concentrations in surface waters, the European Surface Water Watch List has included several antifungal substances [17]. Moreover, another problem arising from unintentional exposure to antifungal agents is the development of resistance, which leads to higher use of multiple substances [18,19].

As a mitigation measure against the increasing environmental concentration of antifungal drugs, research has been performed to assess the effects of sustainable alternatives. Moreover, the development of new antifungal agents could also help to address the mono- and multidrug resistance of diverse fungal species [20]. Much research on yeast fungi is directed towards *Candida* spp. This focus is driven by both their extensively studied medical importance and their growing prevalence and resistance to antifungal drugs [21]. Two novel antifungal agents VT-1161 and T-2307 come as an alternative for the treatment of invasive fungal infections caused by *Candida* spp.

The agent VT-1161, also known as oteseconazole, was approved by the US FDA in April 2022 and is the first approved selective and orally available CYP51 inhibitor [22,23]. By targeting the CYP51 enzyme, the ergosterol biosynthesis is disrupted, leading to the accumulation of toxic sterols and destabilization of the fungal plasma membrane [24]. VT-1161 is effective against fluconazole-resistant *Candida albicans* strains as well as non-albicans species [25].

As for the agent T-2307, it exhibits a novel mechanism of action against antifungal activity by selectively collapsing the mitochondrial membrane potential in yeasts compared to mammalian cells [26,27]. Moreover, this agent is currently under evaluation as clinical data demonstrating its safety and efficacy in humans are currently unavailable [28].

Furthermore, there is currently limited information available on the environmental impacts and ecotoxicity of VT-1161 and T-2307 on non-target species. Therefore, further research is necessary to understand the specific ecotoxicity profiles and potential risks associated with their use in antifungal treatments. In this study, we investigated the ecotoxicological effects of VT-1161 and T-2307 for the first time. We conducted multiple tests to assess their toxicity, including tests for the mobility of the freshwater crustacean *Daphnia magna*, the growth inhibition of the green microalgae *Raphidocelis subcapitata*, the luminescence inhibition of the bioluminescent bacterium *Aliivibrio fischeri*, and the mutagenicity on the bacteria *Salmonella typhimurium* strain TA100.

By examining the ecotoxicity and mutagenicity of VT-1161 and T-2307, this study contributes to understanding their environmental risks. Our findings provide insights into their effects on aquatic organisms, algae, bacteria, and genetic material, aiding in the development of safer and more sustainable antifungal alternatives and mitigating associated environmental impacts.

## 2. Results

### 2.1. Daphnia magna Assay

**Figure 1:**
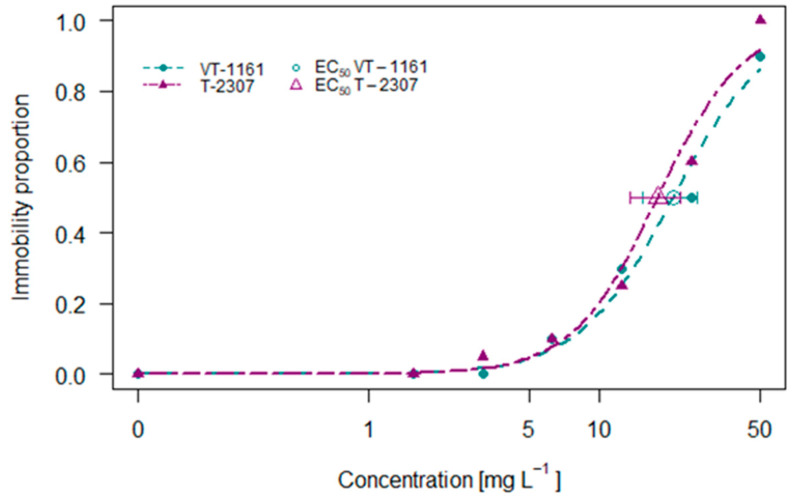
The 48 h concentration–immobility curve of *Daphnia magna* exposed to VT-1161 and T-2307.

The acute toxicity test with *Daphnia magna* exposed to VT-1161 and T-2307 for 48 h revealed a concentration-dependent response in both compounds, as shown in Figure 1. As the concentrations increased, the proportion of immobility also increased for both compounds, reaching an immobility of 100% for VT-1161 and 90% for T-2307 at the concentration of 50 mg L^−1^. The 48 h effective concentrations causing 50% of immobility correspond to 20.94 and 17.93 mg L^−1^ for VT-1161 and T-2307, respectively.

### 2.2. Raphidocelis subcapitata Assay

**Figure 2:**
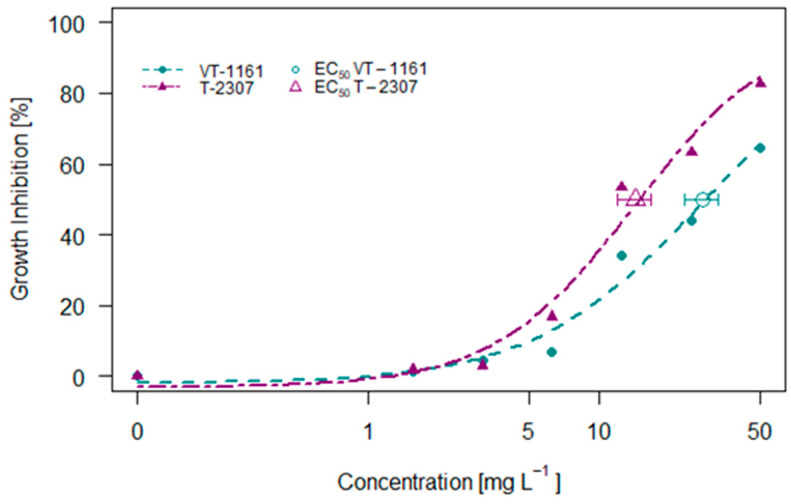
The 72 h concentration–inhibition of growth of *Raphidocelis subcapitata* exposed to VT-1161 and T-2307.

The effects of VT-1161 and T-2307 on the growth of the green algae *Raphidocelis subcapitata* are shown in Figure 2. The inhibitory effects increased with increasing concentration. The compound T-2307 had a higher inhibitory effect, causing around 82% inhibition at 50 mg L^−1^ while VT-1161 caused around 65% inhibition at the same concentration. The effective concentrations causing 50% inhibition correspond to 27.92 and 14.34 mg L^−1^ for VT-1161 and T-2307, respectively.

### 2.3. Aliivibrio fischeri Assay

**Figure 3:**
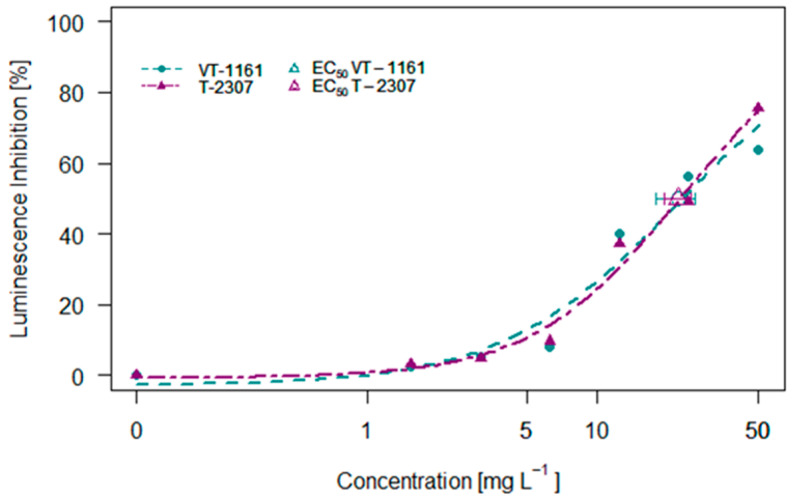
The 30 min concentration–luminescence inhibition of *Aliivibrio fischeri* exposed to VT-1161 and T-2307.

The effects of VT-1161 and T-2307 on the luminescence inhibition of the bacteria *Aliivibrio fischeri* are shown in Figure 3. For this species, increasing concentrations caused higher inhibition of luminescence; however, the effects were maintained below 80% for both compounds. The highest concentration tested of 50 mg L^−1^ caused around 63% and 75% inhibition for VT-1161 and T-2307, respectively. The effective concentrations causing 50% inhibition correspond to 22.39 and 22.58 mg L^−1^ for VT-1161 and T-2307, respectively.

### 2.4. Salmonella typhimurium Assay

**Figure 4:**
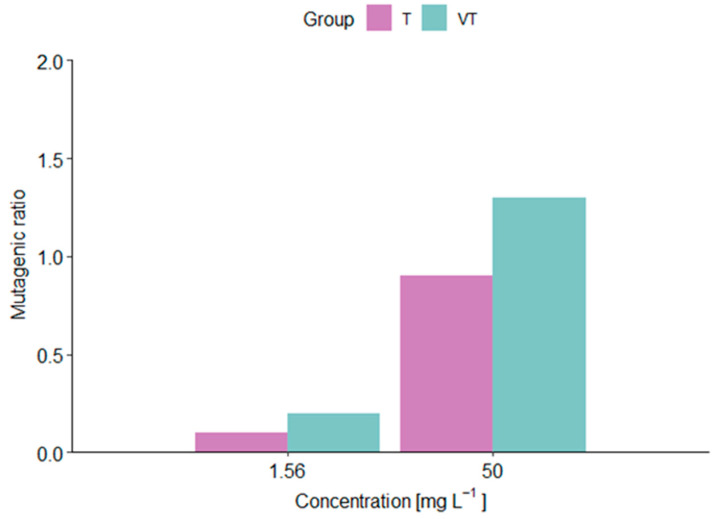
The mutagenic ratio of *Salmonella typhimurium* exposed to VT-1161 and T-2307.

The mutagenicity of VT-1161 and T-2307 to *S. typhimurium* is shown in Figure 4. The mutagenicity ratio was estimated based on the number of revertants (his+). From the two concentrations tested, 1.56 mg L^−1^ had a lower ratio than 50 mg L^−1^. However, both concentrations were below a ratio of 2, resulting in a non-positive mutagenicity induced by the two substances tested.

### 2.5. Hazard Ranking

The hazard ranking of antifungal compounds is crucial for understanding their environmental impact and guiding regulatory decisions. This study utilizes data from existing literature to classify the effective concentrations (EC_50_) of various antifungal compounds according to the Globally Harmonized System of Classification and Labelling of Chemicals (GHS). The effective concentration (EC_50_) is the concentration at which 50% of the maximum effect is observed, serving as a standard measure of toxicity.

Based on the data collected, we have analyzed 37 different exposure scenarios involving several species and antifungal compounds (Table 1). The results indicate a significant variation in toxicity levels across the different compounds and species tested.

Approximately 40% of the effective concentrations (16 out of 37 exposures) fall into this category, characterized by an EC_50_ of ≤1 mg L^−1^. Compounds in this category include clotrimazole, econazole, fluconazole, ketoconazole, and miconazole. These compounds exhibit high toxicity to aquatic organisms such as Daphnia magna and Raphidocelis subcapitata even at very low concentrations.

The second largest category, with 13 scenarios, involves compounds that are classified as toxic with an EC_50_ range between <1 mg L^−1^ and ≤10 mg L^−1^. Compounds like ketoconazole and tebuconazole frequently appear in this category, indicating substantial toxic effects, though not as severe as those in the very toxic category.

Seven scenarios fall under the harmful category, characterized by an EC_50_ between <10 mg L^−1^ and ≤100 mg L^−1^. This classification includes compounds such as VT-1161 and T-2307, which demonstrate moderate toxicity to species like *D. magna* and *A. fischeri*.

Only one scenario is considered not harmful, with an EC_50_ > 100 mg L^−1^. Fluconazole at higher concentrations (530 mg L^−1^) for *D. magna* falls into this category, indicating a significantly lower risk compared to other compounds.

## 3. Discussion

The EC_50_ values obtained for the species tested (*A. fischeri*, *R. subcapitata*, and *D. magna*) for the compounds VT-1161 and T-2307 are indicative of their relative safety compared to other antifungal agents. VT-1161 exhibited EC_50_ values ranging from 20.94 to 27.92 mg L^−1^, while T-2307 had values between 14.34 and 22.58 mg L^−1^.

For instance, clotrimazole’s 48 h EC_50_ for *D. magna* was much lower at 5.14 mg L^−1^ [43] (and an even higher toxicity was reported in [30], with an EC_50_ of 0.020 mg L^−1^). For the compound climbazole, the EC_50_ for *R. subcapitata* (formerly *Pseudokirchneriella subcapitata*) ranged between 0.21 and 1.19 mg L^−1^, while for *D. magna* the EC_50_ was estimated to be 15.99 mg L^−1^ [30]. Furthermore, for ketoconazole’s toxicity, the 48 h EC_50_ for *D. magna* was also estimated at a lower concentration of 1.51 mg L^−1^ [39]. These values were one to two orders of magnitude lower than those obtained for VT-1161 and T-2307, highlighting their lower environmental toxicity.

This lower toxicity is a significant advantage for the potential future use of VT-1161 and T-2307, as they display lower toxicity to non-target organisms. According to the Globally Harmonized System (GHS) classification, both VT-1161 and T-2307 were categorized as harmful (EC_50_ values between 10 mg L^−1^ and 100 mg L^−1^), whereas many other antifungal compounds fall into the very toxic category (EC_50_ values ≤ 1 mg L^−1^).

The high proportion of scenarios classified as very toxic underscores the severe environmental impact of certain antifungal compounds. These findings highlight the need for stringent regulations and the development of safer alternatives. The detailed classification provides a comprehensive overview of the relative hazards associated with each compound, guiding future research and regulatory efforts. The compounds clotrimazole, econazole, and miconazole, which frequently appear in the very toxic category, demand particular attention due to their widespread use and potential for significant ecological disruption. Conversely, compounds like VT-1161 and T-2307, categorized as harmful, represent a moderate risk, yet their impact should not be underestimated.

The lack of mutagenic activity at both tested concentrations is a favorable outcome, suggesting that these compounds do not pose a genetic risk under the conditions evaluated. This is particularly important for the environmental and human health risk assessment of these compounds, as mutagenicity is a critical factor in determining the safety and regulatory status of chemical substances.

While the toxicity of antifungal compounds is crucial, another important factor to consider is their usage, which directly influences their environmental concentrations. Heavy usage of a compound increases its potential to reach the environment. Furthermore, if the compound is not effectively removed by wastewater treatment plants, its probability of reaching the environment rises.

The exposure of non-target species in the environment can lead to the development of resistance and reduce the effectiveness of these compounds against their target species. This is a common issue for many compounds currently on the market and needs to be considered for new compounds yet to be approved. To understand the potential of VT-1161 and T-2307 to reach the environment and expose non-target species, it is important to consider their kinetics and their interactions with other compounds commonly found in wastewater treatment. Currently, pharmacokinetic data are still not available for T-2307 [27]. Further studies should include detailed pharmacokinetic analyses to understand the behavior of these compounds in the environment, their degradation pathways, and their interactions with other chemicals commonly found in wastewater. Such studies will provide a comprehensive understanding of their environmental fate and inform guidelines for their safe use.

## 4. Materials and Methods

### 4.1. Chemicals

T-2307, a synthesized compound from Toyama Chemical Co., Ltd. (Tokyo, Japan), and VT-1161, supplied by Target Mol-tebu-bio, Chemicals Inc. (Mont Belvieu, TX, USA), were used in this study. Stock solutions of the compounds were prepared by dissolving the powders in 5% *v*/*v* artificial freshwater [30] to reach a final concentration of 100 mg L^−1^. Furthermore, the stock solution of T-2307 underwent ultrasonication for 20 min, and the pH was adjusted to 3.0 ± 0.1 using HCl (1 M) to ensure proper dissolution and homogeneity.

### 4.2. Daphnia magna Assay

The immobilization tests with *D. magna* were conducted following ISO guidelines [44] using an in-house culture at the Hygiene Department at the UNINA. Organisms under 24 h old were exposed to concentrations of 1.56, 3.12, 6.25, 12.50, 25, and 50 mg L^−1^ and to a negative control of freshwater media. Each concentration had four replicates containing five organisms each. The exposure was conducted at a temperature of 20 ± 1 °C and under continuous illumination of approx. 1000 lux. Immobilization was assessed at 24 and 48 h after exposure using a stereomicroscope.

### 4.3. Raphidocelis subcapitata Assay

The growth inhibition test with *R. subcapitata* was conducted following ISO guidelines [45] using an in-house culture at the Hygiene Department at the UNINA. Algal cultures were incubated in Erlenmeyer flasks at 20 ± 1 °C on a shaker at 100 rpm and under continuous daylight fluorescent tube illumination of approximately 6300 lux with a 16:8 L:D photoperiod. The initial inoculum concentration was set at 104 cells mL^−1^, exposed to the concentrations of 1.56, 3.12, 6.25, 12.50, 25, and 50 mg L^−1^ and to a negative control of freshwater media. Six replicates were set up for both the control samples and the treatments and were maintained under the same conditions as the algal culture for 72 h.

### 4.4. Aliivibrio fischeri Assay

The luminescence inhibition test was performed on *Allivibrio fischeri* (strain NRRL-B-11177), purchased from WaterTox STD (ECOtest S.L., Valencia, Spain), and performed according to [46]. The luminescence was measured using a Microtox luminometer Model 500, (AZUR Environmental, Carlsbad, CA, USA) equipped with a cell incubated at a constant temperature of 15 ± 1 °C, and readings were taken after 30 min of exposure at a wavelength of 490 nm. The Osmotic Adjustment Solution was added to the media to adapt the marine bacterium to freshwater. The bacteria were exposed to the concentrations of 1.56, 3.12, 6.25, 12.50, 25, and 50 mg L^−1^ and to a negative control.

### 4.5. Salmonella typhimurium Assay

The mutagenicity in *Salmonella typhimurium* strain TA100 was assessed by observing the potential mutations that would allow the bacteria to grow without needing histidine (his-), following the protocol [47]. For the assay, a kit obtained from ECOTOX LDS (Milan, Italy) was used to perform the test. Two exposure concentrations of 1.56 and 50 mg L^−1^ of each compound were used and bromocresol purple was added as a pH indicator. Distilled water served as the negative control and sodium azide as the positive control for TA100 without S9 (human p450).

The plates were incubated at 37 °C for 3 to 5 days and sealed in hermetic plastic bags to avoid contamination. At the end of the exposure period, wells that showed a decrease in pH due to bacterial growth turned yellow and were counted as revertants (his+), indicating a positive result for mutagenicity. Wells that remained purple indicated a negative result (non-mutagenic compound).

### 4.6. Data Analysis

Data were analyzed with R Studio for Windows (Version 2023.06.0) [48]. The effective concentrations causing 50% of the response on the organisms *A. fischeri*, *R. subcapitata*, and *D. magna* were estimated by fitting on dose–response models using the extension package “drc” [49]. For the data analysis of *R. subcapitata* and *A. fischeri* tests, the measurements were normalized to the control to estimate an inhibition percentage.

For the test organism *S. typhimurium*, results were expressed as the mutagenicity ratio. This was calculated by dividing the number of revertants (his+) in the treatments by the number of naturally occurring (negative control). The treatments were considered mutagenic if the mutagenicity ratio was 2 or greater.

Furthermore, multiple fungicide compounds were classified according to the Globally Harmonized System of Classification and Labeling of Chemicals (GHS) [50] to compare the hazard ranking of substances currently on the market and the two substances of this study.

## 5. Conclusions

The experimental results indicate the following effects of the tested compounds: for *Daphnia magna*, the EC50 after 48 h was 20.94 mg L^−1^ for VT-1161 and 17.93 mg L^−1^ for T-2307. In *Raphidocelis subcapitata*, growth inhibition (EC50) after 72 h was 27.92 mg L^−1^ for VT-1161 and 14.34 mg L^−1^ for T-2307. For *Aliivibrio fischeri*, luminescence inhibition (EC50) after 30 min was observed at 20.94 mg L^−1^ for VT-1161 and 22.58 mg L^−1^ for T-2307. These findings illustrate the varying sensitivities of the organisms to the two compounds, with T-2307 exhibiting slightly higher toxicity towards *D. magna* and *R. subcapitata*, while both species maintained the same order of magnitude across all three species tested.

Moreover, the lower toxicity and favorable hazard classification of VT-1161 and T-2307 suggest their potential as safer alternatives for antifungal treatments compared to other compounds documented in the literature. However, it is essential to consider their environmental impact, especially their usage patterns and the efficiency of wastewater treatment processes in removing these compounds. The potential for non-target species to develop resistance underscores the importance of comprehensive environmental risk assessments for new antifungal compounds. Understanding the kinetics and interactions of VT-1161 and T-2307 with other substances in wastewater is crucial for evaluating their environmental safety and ensuring their effectiveness remains uncompromised. Future studies should focus on these aspects to support the responsible development and application of these promising antifungal agents.

## Figures and Tables

**Table 1:** Classification of antifungal compounds following the Globally Harmonized System of Classification and Labelling of Chemicals, according to the effective concentrations causing 50% of the effect.

Compound	Species	Exposure Time (h)	EC_50_ [mg L^−1^]	References	Hazard Ranking
Clotrimazole	*R. subcapitata*	72 h	0.29	[29]	****
	*D. magna*	48 h	0.03	[29]	****
	*Green algae*	96 h	0.13	[30]	****
	*D. magna*	48 h	0.04	[30]	****
	*R. subcapitata*	72 h	0.098	[31]	****
	*D. magna*	48 h	0.02	[31]	****
	*D. magna*	48 h	1.49	[32]	***
Econazole	*D. magna*	48 h	0.24	[33]	****
	*D. magna*	48 h	0.4	[34]	****
	*R. subcapitata*	72 h	1.37	[35]	***
	*R. subcapitata*	72 h	1.37	[36]	***
Fluconazole	*Green algae*	96 h	0.001162	[30]	****
	*D. magna*	48 h	0.002766	[30]	****
	*Green algae*	96 h	55.4	[37]	**
	*D. magna*	48 h	530	[37]	*
Ketoconazole	*Green algae*	96 h	4.36	[30]	***
	*D. magna*	48 h	2.39	[30]	***
	*Green algae*	72 h	0.28	[38]	****
	*D. magna*	48 h	6.57	[38]	***
	*D. magna*	48 h	1.5	[39]	***
	*D. magna*	48 h	6.57	[34]	***
	*R. subcapitata*	72 h	0.28	[36]	****
	*R. subcapitata*	72 h	0.3	[40]	****
Miconazole	*Green algae*	96 h	0.16	[30]	****
	*D. magna*	48 h	0.04	[30]	****
	*Green algae*	72 h	1.35	[38]	***
	*D. magna*	48 h	0.4	[38]	****
	*R. subcapitata*	72 h	1.35	[36]	***
Tebuconazole	*D. magna*	48 h	2.37	[41]	***
	*D. magna*	48 h	6.75	[42]	***
	*R. subcapitata*	72 h	2.09	[42]	***
VT-1161	*D. magna*	48 h	20.94	This study	**
	*R. subcapitata*	72 h	27.92	This study	**
	*A. fischeri*	30 min	20.94	This study	**
T-2307	*D. magna*	48 h	17.93	This study	**
	*R. subcapitata*	72 h	14.34	This study	**
	*A. fischeri*	30 min	22.58	This study	**

Hazard ranking according to effective concentrations values: **** Very toxic (≤1 mg L^−1^); *** Toxic (<1 mg L^−1^ ≤ 10 mg L^−1^); ** Harmful: (<10 mg L^−1^ ≤ 100 mg L^−1^); * Not harmful: (>100 mg L^−1^).

## Data Availability

Data are contained within the article.

## References

[1] Brauer V.S., Rezende C.P., Pessoni A.M., De Paula R.G., Rangappa K.S., Nayaka S.C., Gupta V.K., Almeida F. (2019). Antifungal agents in agriculture: Friends and foes of public health. Biomolecules.

[2] Fisher M.C., Hawkins N.J., Sanglard D., Gurr S.J. (2018). Worldwide emergence of resistance to antifungal drugs challenges human health and food security. Science.

[3] Gross B.N., Steib-Bauert M., Kern W.V., Knoth H., Borde J.P., Krebs S., Hug M.J., Rothe U., Maier L., de With K. (2015). Hospital use of systemic antifungal drugs: A multi-center surveillance update from Germany. Infection.

[4] Markogiannakis A., Korantanis K., Gamaletsou M.N., Samarkos M., Psichogiou M., Daikos G., Sipsas N.V. (2021). Impact of a non-compulsory antifungal stewardship program on overuse and misuse of antifungal agents in a tertiary care hospital. Int. J. Antimicrob. Agents.

[5] Valerio M., Vena A., Bouza E., Reiter N., Viale P., Hochreiter M., Giannella M., Muñoz P., COMIC Study Group (Collaborative Group on Mycosis) (2015). How much European prescribing physicians know about invasive fungal infections management?. BMC Infect. Dis..

[6] Allen D., Wilson D., Drew R., Perfect J. (2015). Azole antifungals: 35 years of invasive fungal infection management. Expert Rev. Anti-Infect. Ther..

[7] Janna H., Scrimshaw M.D., Williams R.J., Churchley J., Sumpter J.P. (2011). From dishwasher to tap? Xenobiotic substances benzotriazole and tolyltriazole in the environment. Environ. Sci. Technol..

[8] FAO FAOSTAT Pesticides Use Dataset. http://www.fao.org/faostat/en/#data/RP.

[9] Bhagat J., Singh N., Nishimura N., Shimada Y. (2021). A comprehensive review on environmental toxicity of azole compounds to fish. Chemosphere.

[10] Boxall A.B. (2004). The environmental side effects of medication: How are human and veterinary medicines in soils and water bodies affecting human and environmental health?. EMBO Rep..

[11] Pacholak A., Burlaga N., Frankowski R., Zgoła-Grześkowiak A., Kaczorek E. (2022). Azole fungicides: (Bio)degradation, transformation products and toxicity elucidation. Sci. Total Environ..

[12] Kahle M., Buerge I.J., Hauser A., Muller M.D., Poiger T. (2008). Azole fungicides: Occurrence and fate in wastewater and surface waters. Environ. Sci. Technol..

[13] Thomas K.V., Hilton M.J. (2004). The occurrence of selected human pharmaceutical compounds in UK estuaries. Mar. Pollut. Bull..

[14] Melefa T.D., Nwani C.D. (2021). Imidazole antifungal drug clotrimazole alters the behavior, brain acetylcholinesterase and oxidative stress biomarkers in African catfish *Clarias gariepinus* (Burchell 1822). Comp. Biochem. Physiol. Part C Toxicol. Pharmacol..

[15] Reno U., Machuca L.M., Regaldo L.M., Murguia M.C., Gagneten A.M. (2021). Environment chemistry: Comparative studies and sublethal ecotoxicity of new antifungals on *Daphnia magna* as model organism. Asian J. Microbiol. Biotechnol. Environ..

[16] Richter E., Wick A., Ternes T.A., Coors A. (2013). Ecotoxicity of climbazole, a fungicide contained in antidandruff shampoo. Environ. Toxicol. Chem..

[17] Gomez Cortes L., Marinov D., Sanseverino I., Navarro Cuenca A., Niegowska M., Porcel Rodriguez E., Stefanelli F., Lettieri T. (2022). Selection of Substances for the 4th Watch List under the Water Framework Directive.

[18] Auberger J., Lass-Flörl C., Aigner M., Clausen J., Gastl G., Nachbaur D. (2012). Invasive fungal breakthrough infections, fungal colonization and emergence of resistant strains in high-risk patients receiving antifungal prophylaxis with posaconazole: Real-life data from a single-centre institutional retrospective observational study. J. Antimicrob. Chemother..

[19] Perlin D.S., Rautemaa-Richardson R., Alastruey-Izquierdo A. (2017). The global problem of antifungal resistance: Prevalence, mechanisms, and management. Lancet Infect. Dis..

[20] Muñoz P., Bouza E. (2016). The current treatment landscape: The need for antifungal stewardship programmes. J. Antimicrob. Chemother..

[21] Maione A., La Pietra A., Siciliano A., Mileo A., De Falco M., de Alteriis E., Guida M., Galdiero E. (2022). The Arylamidine T-2307 as a Novel Treatment for the Prevention and Eradication of *Candida tropicalis* Biofilms. Int. J. Mol. Sci..

[22] De S.K. (2023). Oteseconazole: First approved orally bioavailable and selective CYP51 inhibitor for the treatment of patients with recurrent vulvovaginal candidiasis. Curr. Med. Chem..

[23] Warrilow A., Hull C., Parker J., Garvey E., Hoekstra W., Moore W., Schotzinger R., Kelly D., Kelly S. (2014). The clinical candidate VT-1161 is a highly potent inhibitor of Candida albicans CYP51 but fails to bind the human enzyme. Antimicrob. Agents Chemother..

[24] Hoekstra W.J., Garvey E.P., Moore W.R., Rafferty S.W., Yates C.M., Schotzinger R.J. (2014). Design and optimization of highly-selective fungal CYP51 inhibitors. Bioorganic Med. Chem. Lett..

[25] Ordaya E.E., Clement J., Vergidis P. (2023). The role of novel antifungals in the management of candidiasis: A clinical perspective. Mycopathologia.

[26] Shibata T., Takahashi T., Yamada E., Kimura A., Nishikawa H., Hayakawa H., Nomura N., Mitsuyama J. (2012). T-2307 causes collapse of mitochondrial membrane potential in yeast. Antimicrob. Agents Chemother..

[27] Wiederhold N.P. (2021). Review of T-2307, an investigational agent that causes collapse of fungal mitochondrial membrane potential. J. Fungi.

[28] Nishikawa H., Fukuda Y., Mitsuyama J., Tashiro M., Tanaka A., Takazono T., Saijo T., Yamamoto K., Nakamura S., Imamura Y. (2017). In vitro and in vivo antifungal activities of T-2307, a novel arylamidine, against Cryptococcus gattii: An emerging fungal pathogen. J. Antimicrob. Chemother..

[29] Gonçalves N.P., del Puerto O., Medana C., Calza P., Roslev P. (2021). Degradation of the antifungal pharmaceutical clotrimazole by UVC and vacuum-UV irradiation: Kinetics, transformation products and attenuation of toxicity. J. Environ. Chem. Eng..

[30] Chen Z.-F., Ying G.-G. (2015). Occurrence, fate and ecological risk of five typical azole fungicides as therapeutic and personal care products in the environment: A review. Environ. Int..

[31] Morais S.A., Delerue-Matos C., Gabarrell X. (2014). An uncertainty and sensitivity analysis applied to the prioritisation of pharmaceuticals as surface water contaminants from wastewater treatment plant direct emissions. Sci. Total Environ..

[32] Yuan S., Liang C., Li W., Letcher R.J., Liu C. (2021). A comprehensive system for detection of behavioral change of *D. magna* exposed to various chemicals. J. Hazard. Mater..

[33] Valimaña-Traverso J., Amariei G., Boltes K., García M.Á., Marina M.L. (2019). Enantiomer stability and combined toxicity of duloxetine and econazole on *Daphnia magna* using real concentrations determined by capillary electrophoresis. Sci. Total Environ..

[34] Tkaczyk A., Bownik A., Dudka J., Kowal K., Ślaska B. (2021). *Daphnia magna* model in the toxicity assessment of pharmaceuticals: A review. Sci. Total Environ..

[35] Valimaña-Traverso J., Amariei G., Boltes K., García M.Á., Marina M.L. (2019). Stability and toxicity studies for duloxetine and econazole on *Spirodela polyrhiza* using chiral capillary electrophoresis. J. Hazard. Mater..

[36] Villain J., Minguez L., Halm-Lemeille M.-P., Durrieu G., Bureau R. (2016). Acute toxicities of pharmaceuticals toward green algae. mode of action, biopharmaceutical drug disposition classification system and quantile regression models. Ecotoxicol. Environ. Saf..

[37] Cai W., Ye P., Yang B., Shi Z., Xiong Q., Gao F., Liu Y., Zhao J., Ying G. (2021). Biodegradation of typical azole fungicides in activated sludge under aerobic conditions. J. Environ. Sci..

[38] Minguez L., Pedelucq J., Farcy E., Ballandonne C., Budzinski H., Halm-Lemeille M.-P. (2016). Toxicities of 48 pharmaceuticals and their freshwater and marine environmental assessment in northwestern France. Environ. Sci. Pollut. Res..

[39] Haeba M.H., Hilscherová K., Mazurová E., Bláha L. (2008). Selected endocrine disrupting compounds (vinclozolin, flutamide, ketoconazole and dicofol): Effects on survival, occurrence of males, growth, molting and reproduction of *Daphnia magna*. Environ. Sci. Pollut. Res..

[40] Yousefi-Ahmadipour A., Bozorgi-Koshalshahi M., Mogharabi M., Amini M., Ghazi-Khansari M., Faramarzi M.A. (2016). Laccase-catalyzed treatment of ketoconazole, identification of biotransformed metabolites, determination of kinetic parameters, and evaluation of micro-toxicity. J. Mol. Catal. B Enzym..

[41] Tofan L., Niță V., Nenciu M., Coatu V., Lazăr L., Damir N., Vasile D., Popoviciu D.R., Brotea A.-G., Curtean-Bănăduc A.M. (2023). Multiple assays on non-target organisms to determine the risk of acute environmental toxicity in tebuconazole-based fungicides widely used in the black sea coastal area. Toxics.

[42] Del Puerto O., Gonçalves N.P., Medana C., Prevot A.B., Roslev P. (2022). Attenuation of toxicity and occurrence of degradation products of the fungicide tebuconazole after combined vacuum UV and UVC treatment of drinking water. Environ. Sci. Pollut. Res..

[43] Vieira M., Soares A.M., Nunes B. (2019). Biomarker-based assessment of the toxicity of the antifungal clotrimazol to the microcrustacean *Daphnia magna*. Environ. Toxicol. Pharmacol..

[44] (2012). Water Quality—Determination of the Inhibition of the Mobility of *Daphnia magna* Straus (Cladocera, Crustacea)—Acute Toxicity Test.

[45] (2012). Water Quality—Fresh Water Algal Growth Inhibition Test with Unicellular Green Algae.

[46] (2007). Water Quality—Determination of the Inhibitory Effect of Water Samples on the Light Emission of *Vibrio fischeri* (Luminescent Bacteria Test).

[47] Ames B.N., McCann J., Yamasaki E. (1975). Methods for detecting carcinogens and mutagens with the salmonella/mammalian-microsome mutagenicity test. Mutat. Res./Environ. Mutagen..

[48] RStudio Team (2023). RStudio: Integrated Development for R.

[49] Ritz C., Baty F., Streibig J.C., Gerhard D. (2015). Dose-response analysis using R. PLoS ONE.

[50] United Nations (2002). Globally Harmonized System of Classification and Labelling of Chemicals (GHS).

